# In Vivo Targeting of CXCR4—New Horizons

**DOI:** 10.3390/cancers13235920

**Published:** 2021-11-25

**Authors:** Margret Schottelius, Ken Herrmann, Constantin Lapa

**Affiliations:** 1 Translational Radiopharmaceutical Sciences, Department of Nuclear Medicine and of Oncology, Centre Hospitalier Universitaire Vaudois (CHUV), University of Lausanne (UNIL), 1011 Lausanne, Switzerland; 2 Department of Nuclear Medicine, German Cancer Consortium (DKTK)-University Hospital Essen, University of Duisburg-Essen, 45147 Essen, Germany; ken.herrmann@uk-essen.de; 3 Nuclear Medicine, Medical Faculty, University of Augsburg, 86156 Augsburg, Germany

**Keywords:** CXCR4, cancer, inflammation, molecular imaging, PET, SPECT, radioligand therapy

## Abstract

**Simple Summary:**

Beyond its pre-eminent role in the context of tumor cell growth as well as metastasis, the C-X-C motif chemokine receptor 4 (CXCR4) is a key player in the orchestration of inflammatory responses to inflammatory stimuli. This review therefore particularly focuses on summarizing the current knowledge on non-invasive imaging of tissue infiltration with CXCR4-expressing immune cells in the context of diverse inflammatory conditions using positron emission tomography. An overview of the current clinical and preclinical approaches in this context is provided, and the recent advances in the development of dedicated, high-end CXCR4-targeted imaging tools and theranostic agents are discussed.

**Abstract:**

Given its pre-eminent role in the context of tumor cell growth as well as metastasis, the C-X-C motif chemokine receptor 4 (CXCR4) has attracted a lot of interest in the field of nuclear oncology, and clinical evidence on the high potential of CXCR4-targeted theranostics is constantly accumulating. Additionally, since CXCR4 also represents a key player in the orchestration of inflammatory responses to inflammatory stimuli, based on its expression on a variety of pro- and anti-inflammatory immune cells (e.g., macrophages and T-cells), CXCR4-targeted inflammation imaging has recently gained considerable attention. Therefore, after briefly summarizing the current clinical status quo of CXCR4-targeted theranostics in cancer, this review primarily focuses on imaging of a broad spectrum of inflammatory diseases via the quantification of tissue infiltration with CXCR4-expressing immune cells. An up-to-date overview of the ongoing preclinical and clinical efforts to visualize inflammation and its resolution over time is provided, and the predictive value of the CXCR4-associated imaging signal for disease outcome is discussed. Since the sensitivity and specificity of CXCR4-targeted immune cell imaging greatly relies on the availability of suitable, tailored imaging probes, recent developments in the field of CXCR4-targeted imaging agents for various applications are also addressed.

## 1. Introduction

Given its pre-eminent role in the context of tumor cell growth as well as metastasis, the C-X-C motif chemokine receptor 4 (CXCR4) has attracted a lot of interest in nuclear oncology. Powerful imaging agents and a theranostic concept targeting CXCR4 have evolved during the last decade, and their increasing clinical impact is reflected by an eleven-fold increase in the number of publications per year (PubMed search for “CXCR4 imaging PET cancer”) during the last decade (2011–2020) and highlighted in several comprehensive reviews [1,2,3,4].

Of note, CXCR4 is also endogenously expressed on various pro- and anti-inflammatory immune cells, with particularly high expression levels on macrophages and T cells, making the receptor one of the key players in the orchestration of inflammatory responses of the body to a variety of local and systemic inflammatory stimuli. Imaging inflammation (i.e., infiltration of tissues by CXCR4-expressing immune cells) by targeting CXCR4 has therefore become another very vibrant field of preclinical and clinical research. After a short summary of the recent clinical advances in CXCR4-targeted imaging and radioligand therapy (RLT) in cancer, this review will therefore particularly highlight the increasing body of evidence of the clinically relevant role of CXCR4 imaging in the context of inflammatory conditions. Important recent findings in preclinical studies in this field as well as the very recent developments of innovative and promising new tracer concepts will also be addressed. 

## 2. CXCR4-Targeted Theranostics in Cancer and Its Limitations

### 2.1. CXCR4-Targeted PET Imaging

Although many underlying mechanisms and their implications for disease progression are not elucidated yet, most tumors import a worsening prognosis with increasing CXCR4 expression, which is known to play an important role in both tumor cell proliferation and metastasis [5]. Non-invasive imaging of receptor expression has become feasible through the introduction of radiolabeled ligands that allow for whole-body SPECT or PET [2], with the theranostic concept based on the cyclic pentapeptide tracers [^68^Ga]Pentixafor and [^177^Lu]Pentixather being the farthest advanced into clinical practice. 

Since CXCR4 expression is generally high among hematological malignancies, including non-Hodgkin lymphoma (NHL), multiple myeloma (MM), chronic lymphocytic leukemia (CLL) and acute myeloid leukemia (AML), most experience with CXCR4-directed PET imaging is based on patients with these diseases [6].

In MM, CXCR4-targeted PET imaging using [^68^Ga]Pentixafor has been demonstrated to yield complementary as well as superior results in both newly diagnosed as well as advanced disease compared to [^18^F]FDG-PET [7,8,9]. Of note, marked inter- as well as intra-individual differences of receptor expression as a sign of clonal heterogeneity [10] and dynamic receptor regulation [11] have been observed, requiring further research to gain a deeper understanding of the underlying biology. In AML, where the CXCR4/CXCL12 axis is crucially involved in attraction of leukemic blasts into the protective bone marrow (BM) niche, [^68^Ga]Pentixafor PET was able to identify CXCR4-positive patients in roughly 50% of cases [12]. Another study revealed that the BM involvement of CLL is associated with significantly higher tracer uptake as compared to healthy controls [13].

Additionally, CXCR4 has recently emerged as an interesting molecular target in marginal zone lymphoma [14,15], gastric mucosa-associated lymphoid tissue (MALT) lymphoma [16], mantle cell lymphoma [17], myeloproliferative neoplasms [18] as well as primary lymphoma of the central nervous system [19]. In Waldenström macroglobulinemia/lymphoplasmacytic lymphoma, a disease in which about 40% of patients have mutations in the CXCR4 gene, preliminary results with non-invasive PET imaging using [^68^Ga]Pentixafor have demonstrated promising results in both staging and therapy response assessment. Of note, available data suggest potential superiority over [^18^F]FDG-PET [20,21,22].

Beyond hematologic malignancies, CXCR4 overexpression has been described in various solid cancers [23]. As in the case of the former, enhanced CXCR4 expression in solid tumors characterizes more aggressive disease and portends an unfavorable prognosis [24]. This is also supported by a study that demonstrated an inverse correlation between tumor differentiation and CXCR4 expression, as assessed by immunohistochemistry of surgical samples in neuroendocrine neoplasms [25].

However, in contrast to the sometimes-pronounced CXCR4 expression observed in ex vivo immunohistochemistry studies using tumor biopsies, the corresponding in vivo [^68^Ga]Pentixafor PET/CT studies demonstrated a very heterogeneous, often modest, and in some cases even absent CXCR4-mediated tracer uptake in solid cancers [26,27,28,29], limiting the broad diagnostic application of this imaging approach. To date, only few solid tumor entities have shown pronounced overexpression of CXCR4, as assessed by PET/CT. For example, some studies have reported intense overexpression of CXCR4 in patients with SCLC [30,31,32], and Bluemel et al. demonstrated feasibility of CXCR4-directed PET/CT imaging in patients with advanced adrenocortical cancer. In their initial patient cohort, about 70% of subjects demonstrated sufficient tumoral [^68^Ga]Pentixafor uptake to qualify for potential CXCR4-directed radioligand therapy (RLT) [33].

### 2.2. CXCR4-Targeted Radioligand Therapy

Pentixather, the therapeutic counterpart of Pentixafor, can be labeled with various radionuclides for CXCR4-directed radioligand therapy (RLT) [34]. In patient-derived xenograft mouse models of acute lymphocytic leukemia (ALL) and AML [35], a significant eradication of leukemic blasts has been recorded. These encouraging results led to the translation to the human setting, with three patients with refractory AML successfully undergoing CXCR4-directed RLT.

To date, more than 70 chemokine receptor-directed radioligand therapies using [^177^Lu/^90^Y]Pentixather have been conducted. Given the high specificity of the therapeutic compound for human CXCR4, the therapy is generally safe and well-tolerated [36]. So far, the largest studies have examined the use of RLT in advanced stage, heavily pretreated MM patients. Although initial response rates were high, no substantial overall survival benefit could be observed [37,38]. Another pilot investigation showed encouraging results using [^177^Lu/^90^Y]Pentixather RLT in diffuse large B cell lymphoma [39]. Currently, one prospective trial for CXCR4-directed RLT, which will investigate the activity and tolerable dose and side effects of [^177^Lu/^90^Y]Pentixather RLT in patients with MM or NHL (COLPRIT trial, EudraCT 2015-001817-28), is in preparation.

Due to its effect on the BM, CXCR4-directed RLT usually requires subsequent HSC transplantation. However, in hematologic diseases, profound myeloablation by CXCR4-targeted RLT prior to autologous or allogeneic hematopoietic transplantation is a desired effect that has already been enhanced by the addition of anti-cluster of differentiation 66 (CD66)-directed or anti-CD20-directed radioimmunotherapy in some cases [39].

In contrast, in solid malignancies, in which hematopoietic cell transplantation is not an established and suitable approach, such myeloablation is certainly of concern. [^177^Lu/^90^Y]Pentixather RLT without stem cell rescue might be technically feasible for tumors with pronounced receptor overexpression, as exemplified by adrenocortical cancer or SCLC, but further development and prospective investigations are definitely required.

## 3. Beyond Cancer: CXCR4-Targeted Imaging of Immune Cell Infiltrates

Since CXCR4 is also expressed on a variety of pro- and anti-inflammatory immune cells, with a pronounced overexpression on macrophages and T cells, CXCR4-directed molecular imaging provides the opportunity to gain insight into the highly sophisticated and orchestrated role of chemokines and their receptors in inflammatory processes [4,40]. Of note, due to the relatively broad spectrum of immune cells expressing CXCR4, imaging of inflammatory processes using CXCR4-targeted tracers such as [^68^Ga]Pentixafor will invariably depict the global infiltration of inflamed tissue with pro- and anti-inflammatory immune cells, without the possibility to specifically attribute the imaging signal to a specific immune cell subtype (except by immunohistochemical analyses) [41].

### 3.1. Cardiovascular Diseases

Pilot studies investigating CXCR4-directed PET imaging in the setting of acute myocardial infarction (AMI) demonstrated high [^68^Ga]Pentixafor uptake in the infarcted myocardium [42,43], which correlated with the presence of pro-inflammatory cells in the ischemic area [42]. Another investigation revealed an association between CXCR4 expression in the BM and the severity of the systemic inflammatory response, and suggested that AMI patients with initially high myocardial tracer uptake developed less scar tissue in the infarcted area and presented with a better functional outcome at follow-up [44]. However, the latter findings are in contradiction with a recent pre-clinical study in post-AMI mice that provided evidence that imaging-guided on-peak CXCR4 inhibition (three days after AMI) lowered the risk of ventricular rupture, accelerated resolution of post-infarct inflammation and improved the outcome (please also see Section 4) [45]. In line with this finding, beneficial effects of CXCR4 blockade by attenuated inflammatory gene expression via regulatory T cells (Tregs) have been reported [46]. CXCR4-positive cardio-protective Tregs were found to accumulate in infarcted myocardium and mediastinal lymph nodes in a mouse model, as well as in patients after AMI, interpreted as a sign of pro-healing autoimmunity [47]. More research to deepen our understanding of the spatial and temporal orchestration of CXCR4 expression of the various cell types involved in AMI and to explore potential therapeutic interventions is highly warranted.

### 3.2. Inflammation Imaging

Recently, a study in 72 lymphoma patients found that [^68^Ga]Pentixafor PET/MRI was suitable to visualize inflammation within human carotid plaques, with histological evidence for co-localization of CXCR4 and CD68 [48], thereby confirming previous results of chemokine receptor overexpression in macrophage-rich plaques in a rabbit model of atherosclerosis [49]. In addition, two independent studies using [^68^Ga]Pentixafor PET/CT demonstrated an association between cardiovascular risk factors and CXCR4 expression within atherosclerotic plaques on a per-patient basis [50,51]. Derlin et al. investigated CXCR4 expression in the coronary arteries of AMI patients after stent-based reperfusion and observed the highest [^68^Ga]Pentixafor uptake in the culprit lesions, which the authors ascribed to inflammatory changes and/or stent-induced injury [52]. In another study in patients undergoing CXCR4-directed RLT for hematologic malignancy, Li et al. were able to show an additional anti-inflammatory therapeutic effect on atherosclerotic plaques [53].

While most of the PET signal in [^68^Ga]Pentixafor PET is considered to originate from macrophages, the variety of different cell types (T cells, B cells and/or progenitor cells) expressing CXCR4 on their surface adds to the complexity of the underlying biology [54]. Furthermore, the CXCR4/CXCL12 axis seems to exert both athero-protective as well as atherogenic, pro-inflammatory effects [55]. This could explain the results of a human carotid plaque study that showed CXCR4 overexpression in both stable and unstable atherosclerotic plaques, with the highest receptor expression on macrophages and macrophage-derived foam cells [56]. In addition, in a recent retrospective comparison of oncologic patients undergoing imaging with [^68^Ga]Pentixafor and [^18^F]FDG PET/CT for staging purposes, imaging results obtained with both tracers showed only a weak correlation (r = 0.28; *p* < 0.01). However, CXCR4-directed imaging was able to identify more lesions, and the degree of plaque calcification was found to correlate negatively with [^68^Ga]Pentixafor uptake [57]. While these preliminary results are interesting and hint towards a suitability of [^68^Ga]Pentixafor PET for differentiating between vulnerable and stable plaques, future research to further investigate CXCR4 biology in atherosclerosis and its clinical implications is highly warranted.

Another highly relevant clinical application of CXCR4-targeted inflammation imaging is the non-invasive assessment of disease activity in idiopathic pulmonary fibrosis (IPF) [58]. In a study with 28 subjects, high CXCR4 expression was primarily observed in macrophages, lymphocytes and epithelial cells in areas of vast fibrotic remodeling. [^68^Ga]Pentixafor PET/CT was found to sensitively detect early changes in pulmonary CXCR4 expression upon treatment with an antifibrotic drug, with a decrease in [^68^Ga]Pentixafor uptake 6–12 weeks after treatment initiation as the only independent predictor of long-term outcome. CXCR4 expression in IPF may thus serve as a valuable biomarker for individualized antifibrotic therapies.

### 3.3. Infection Imaging

Non-invasive imaging of CXCR4 with [^68^Ga]Pentixafor PET/CT has also been investigated in infectious diseases. In a pilot study of 29 patients with suspected chronic osteomyelitis, Bouter et al. targeted the elevated CXCR4 expression on T cells to successfully visualize inflammatory activity, with superior diagnostic accuracy of [^68^Ga]Pentixafor PET/CT for detection of chronic bone infections as compared to granulocyte-directed ^99m^Tc-besilesomab and ^99m^Tc-labeled white blood cells [59,60]. As another example, in a pilot investigation of 13 patients with complicated urinary tract infections after kidney transplantation, infectious foci could successfully be detected by imaging leukocyte infiltration using [^68^Ga]Pentixafor PET/MRI [61].

**Figure 1:**
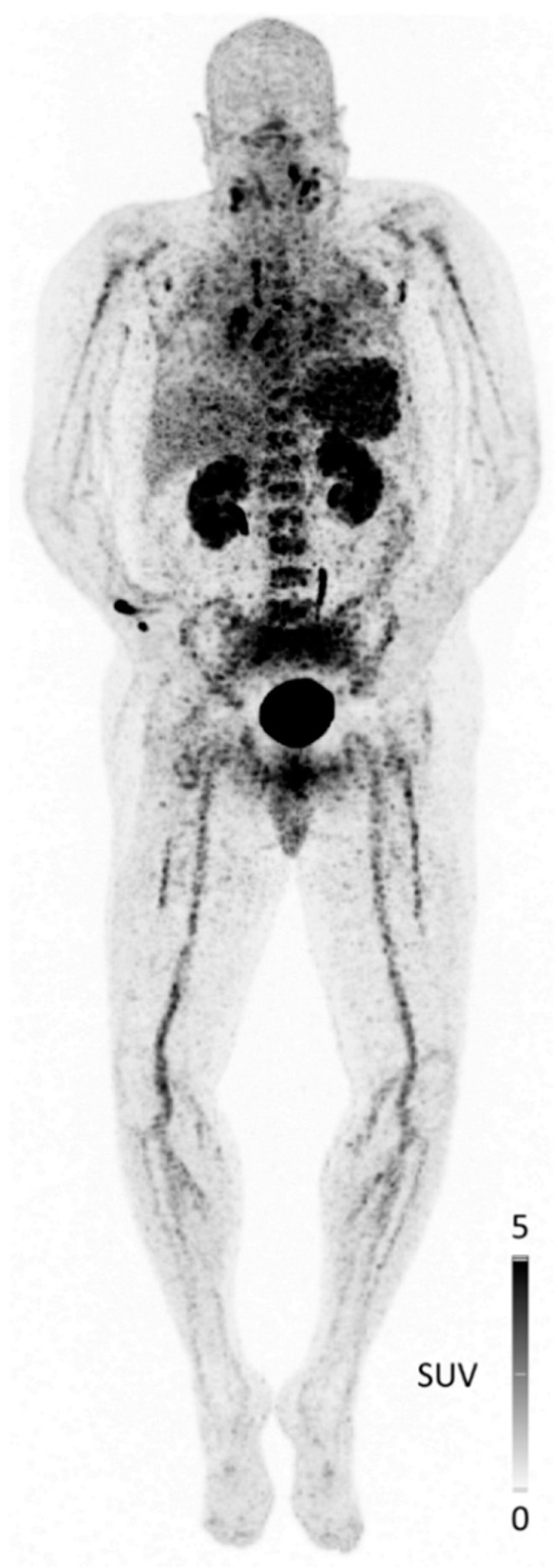
Maximum intensity projection of CXCR4-directed PET/CT with [^68^Ga]Pentixafor in a 67-year-old patient with acute COVID-19 infection. Beyond bilateral pneumonia with reactive hilar and mediastinal lymph nodes, inflammatory foci in the lymphoid tissue of the neck could be depicted. In addition, the reactive activation of both bone marrow and spleen is visualized. The patient’s condition deteriorated quickly after imaging and he was transferred to the ICU on the day after PET/CT [C. Lapa, unpublished data].

Lastly, in the context of the recent COVID-19 pandemic, CXCR4-directed imaging of both infectious sites and reactive systemic responses of the reticulo-endothelial system has been initiated (Figure 1). In a first patient with acute COVID-19 symptoms, bilateral pneumonia with reactive hilar and mediastinal lymph nodes as well as inflammatory foci in the lymphoid tissue of the neck were clearly visualized by [^68^Ga]Pentixafor PET.

Furthermore, reactive activation of both bone marrow and spleen as a response of the immune system to the viral infection was reflected by increased tracer uptake in these organs. Although preliminary in nature, these data demonstrate the potential of [^68^Ga]Pentixafor PET for imaging of local immune cell infiltration as a result of viral infection, and further studies to corroborate these findings are underway.

## 4. Imaging of CXCR4-Positive Immune Cells in Preclinical Models

Interestingly, as opposed to the many examples of successful in vivo imaging of CXCR4-positive immune cell infiltrates in various clinical scenarios of inflammation and infection summarized above, preclinical studies in this context are rather rare. On the one hand, this is certainly due to the limitations encountered by imaging probes targeted to the murine CXCR4 receptor. As described in more detail in the next section, high mCXCR4 expression in mouse tissues such as liver, lung and spleen leads to considerable activity accumulation in these organs, thus obscuring the oftentimes weak but significant signal arising from immune cell infiltration of the heart, muscle or aortic plaques in mice. On the other hand, [^68^Ga]Pentixafor, the only currently available tracer with negligible background accumulation in mCXCR4-positive organs, is highly selective for hCXCR4. With a mCXCR4 affinity of >1 μM [62], [^68^Ga]Pentixafor can only provide a very weak mCXCR4-specific signal, challenging its usefulness for investigating CXCR4-mediated pathological processes in mouse models.

That this is nevertheless feasible in some instances, however, has been shown in several preclinical studies investigating the dynamics and involvement of CXCR4 expression in cardiac diseases [63,64,65,66]. In a mouse model of acute myocardial infarction, [^68^Ga]Pentixafor PET allowed identification of an increased inflammatory leucocyte content in the injured ventricle at 1 and 3 days after infarction. Additionally, a delayed resolution of myocardial inflammation (no decreased [^68^Ga]Pentixafor uptake in the injured myocardium between days 1 and 3 after infarction) was shown to be a negative prognostic marker for survival [45]. Work from the same group also demonstrated the utility of [^68^Ga]Pentixafor PET for detecting diffuse myocardial inflammation in a mouse model of pressure overload heart failure [63] and was able to reveal the tight inflammatory interaction between the myocardium and the kidneys after acute myocardial inflammation [66]. In the latter study, the gradual decline in [^68^Ga]Pentixafor uptake in injured myocardium and the kidneys over time revealed identical kinetics of post-infarction resolution of inflammation.

In the context of atherosclerosis, CXCR4-targeted imaging of vulnerable plaques via [^68^Ga]Pentixafor PET has already found its way into clinical application (see Section 3.2). Its superiority for accurately differentiating plaque phenotypes compared to other tracers has been very recently shown in an ex vivo autoradiography study using sections of human carotid plaques [67]. In this study, tracers targeted to different molecular markers for plaque imaging (sst_2_, leukocyte function-associated antigen-1 (LFA-1), folate receptor (FR) and CXCR4) were comparatively evaluated. Here, differential CXCR4-mediated accumulation of [^67^Ga]Pentixafor was shown to allow the most precise differentiation between early, stable (calcified) and vulnerable plaques. Another very recent study used ^64^Cu-labeled viral macrophage inflammatory protein, [^64^Cu]DOTA-vMIP-II (see entry 22, Table 1), for imaging atherosclerosis in Apoe^-/-^ mice. This study revealed that, in addition to the well-documented accumulation of CXCR4-targeted probes in macrophages and foam cells in atherosclerotic plaques [49], a large proportion of [^64^Cu]DOTA-vMIP-II accumulation in atherosclerotic lesions was mediated by CXCR4-expressing plaque endothelial cells (ECs). This, and the finding that plaque regression was accompanied by a loss of CXCR4 on aortic endothelium, led to the conclusion that high CXCR4 expression on plaque ECs may serve as a molecular marker for endothelial vulnerability/injury [68]. 

Two other very recent preclinical studies using a small-molecule tracer (entry 6, Table 1) and N-[^11^C]methyl-AMD3465 as PET imaging agents further highlight the relevance of CXCR4 as a target for immune cell imaging [74,88]. In a model of carrageenan-induced inflammation, significantly higher accumulation of the benzenesulfonamide ligand [^18^F]5 was observed in the paw edema lesion as compared to the control paw, indicative of the inflammation-induced infiltration with CXCR4-positive immune cells [74]. In the context of immuno-oncology, an accurate assessment of tumor immune cell infiltration as a response to immunomodulatory cancer treatments is of pivotal importance. That this can be achieved by CXCR4-targeted immune cell imaging has been recently demonstrated preclinically [88]. Tumors of xenograft-bearing mice were either treated with single-fraction radiotherapy or single-fraction radiotherapy, followed by immunization with a single dose of a virus-based antitumor vaccine. Radiation therapy alone already led to a 3.5-fold increase of N-[^11^C]methyl-AMD3465 uptake in the treated tumor compared to untreated controls, and this effect was further enhanced by immunization. This therapy-induced tumor infiltration with CXCR4-expressing immune cells was efficiently inhibited by daily administration of AMD3100 on the days following irradiation.

Although probably only visualizing the tip of the iceberg concerning the multitude of potentially relevant applications, these examples impressively demonstrate the potential of CXCR4-directed imaging of immune cells and their trafficking to sites of inflammation, and an increasing number of preclinical and clinical studies in this context can be anticipated for the coming years.

## 5. CXCR4-Targeted Imaging Agents: An Ever-Evolving Field

**Figure 2:**
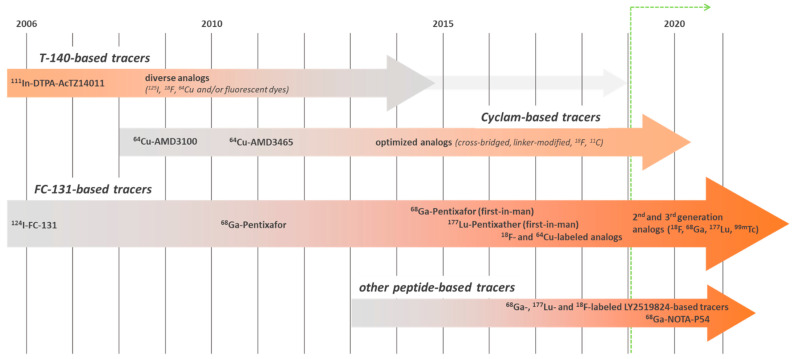
Historical evolution and relevance of the development of CXCR4-targeted imaging probes based on different chemical scaffolds (green line indicates the timeframe covered in this review).

Based on the identification of the first highly potent, synthetic CXCR4-targeted anti-HIV antagonists in the late 1990s [89,90], and fueled by the recognition of the important role of CXCR4 in cancer growth, progression and metastasis in the early 2000s [91], the development of CXCR4-targeted imaging agents has been a field of highly active research since the mid-2000s (Figure 2). Several excellent reviews on the progressive advances in the design of CXCR4-directed nuclear, fluorescent and hybrid imaging agents have been published over the years [1,3,92,93,94]. Since they already provide an impressive and comprehensive overview of the historical evolution of the different tracer concepts (summarized in Figure 2), only very recent (2019 and later) advances and developments will be discussed in this review.

As evident from the summary in Figure 2, the relevance of tracers based on the T140-peptide scaffold has been decreasing during the last decade, primarily based on inherent physicochemical characteristics of the peptide backbone (highly cationic nature). Due to inevitably high non-specific background accumulation in the excretion organs, in particular in the liver, T140-based ligands such as [^68^Ga]NOTA-NFB imaging deliver up to ten times higher effective doses in patients compared to [^68^Ga]Pentixafor [95,96], challenging their potential for further clinical application. Thus, not surprisingly, only one novel T140—based compound (for SPECT imaging) has been published since 2019 (entry 21, Table 1), displaying the same characteristics as previous probes based on this scaffold [85].

**Figure 3:**
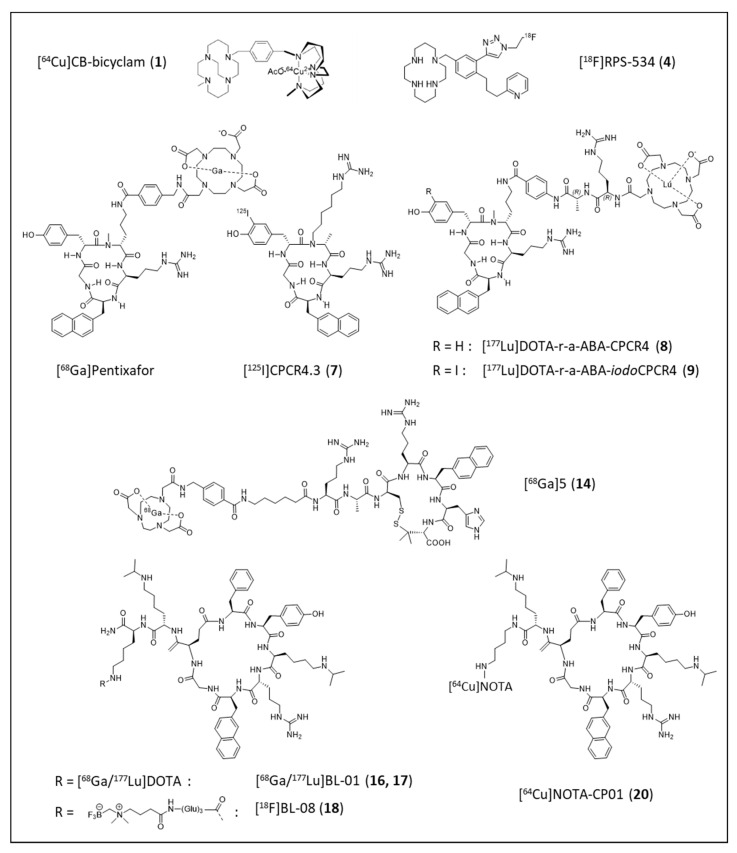
Chemical structures of the clinically used CXCR4 imaging agent [^68^Ga]Pentixafor and of selected novel CXCR4-targeted tracers under preclinical evaluation. Bold numbers in parentheses represent the entry number of the respective compound in Table 1.

In contrast, the development of optimized mono- and bi-cyclam-based tracers (entries 1–5, Table 1) for PET and SPECT imaging is still ongoing. As summarized in Table 1, reasonable to high tumor/background ratios and CXCR4-specificity of tumor uptake were found for several novel ligands. Unfortunately, however, their overall pharmacokinetics are generally vitiated by extensive activity accumulation in the excretion organs. A large proportion of the pronounced hepatic uptake of radiolabeled (mono/bi)cyclams in mice, however, has been shown to be blockable by co-injection of the corresponding unlabeled competitor [69,71,72], a finding that is in line with the documented expression of mCXCR4 in mouse liver [69,97,98] and thus indicative of a significant contribution of CXCR4-specific tracer accumulation. This effect has also been investigated in more detail during the evaluation of the cross-bridged bi-cyclam [^64^Cu]CB-bi-cyclam (entry 1, Table 1, and Figure 3). Here, hepatic [^64^Cu]CB-bi-cyclam uptake was successfully blocked by pre-dosing with 5 mg/kg of Cu-CB-bi-cyclam (IC_50_ = 8 nM) or Cu_2_-CB-bi-cyclam (IC_50_ = 3 nM), whereas the low-affinity “standard” competitors AMD3100 (IC_50_ = 204 nM) and AMD3465 (IC_50_ = 114 nM) were not able to competitively displace the radioligand in vivo [69]. As opposed to the first-generation, non-stabilized AMD-based tracer [^64^Cu]AMD 3100, for which extensive hepatic tracer accumulation was not only observed in mice [99], but also in humans [100], an effect that is due to excessive transchelation of ^64^Cu in the liver and general metabolic instability of the complex, [^64^Cu]CB-bi-cyclam shows excellent in vivo stability. Thus, for this analog, only minimal tracer accumulation in the human liver may be anticipated, strongly supporting its suitability for clinical translation.

As found during the in vivo evaluation of the mono-cyclam analog [^18^F]MCFB (entry 2, Table 1), organic cation transporters (OCT1, OCT2 and OCT3) also seem to be partly involved in the uptake of the cationic radiolabeled mono/bi-cyclams into the liver. When an excess of the OCT-substrate metformin was co-administered with [^18^F]MCFB, a reduction of tracer accumulation in the liver by approximately 25% was observed, with a less pronounced effect in the kidneys [70]. Another interesting effect that was observed during the evaluation of different radio-iodinated and radio-brominated mono-cyclam analogs [73] is the dependence of their general pharmacokinetics and targeting efficiency on the radio-labeling site. Of the two radio-brominated isomers investigated, [^76^Br]HZ270-1 (entry 5, Table 1, 2-[^76^Br]bromo-analog) showed distinctly lower background accumulation in all organs, and a doubled tumor accumulation compared to the corresponding 3-[^76^Br]bromo-analog, highlighting the importance of establishing detailed structure activity relationships in tracer development.

As exemplified by the sheer number of novel analogs published since 2019 (entries 7–20, Table 1), small cyclic peptides (5 to 8 amino acids) play a prominent role as scaffolds for novel CXCR4-targeted imaging agents [62,75,76,77,78,79,80,81,82,83,84].

**Figure 4:**
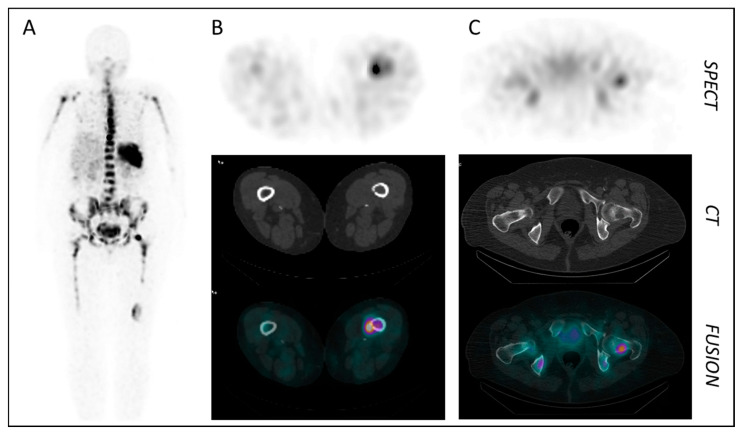
CXCR4-targeted SPECT/CT imaging in a patient with multiple myeloma (MM) using [^99m^Tc]PentixaSPECT. (**A**) MIP at 3 h p.i. (**B**,**C**) Axial SPECT, CT and fusion images of intramedullary MM lesions in the femur (**B**) and the femoral head (**C**) (3 h p.i.) [78].

Based on the clinical success of [^68^Ga]Pentixafor/[^177^Lu]Pentixather as a first CXCR4-targeted theranostic pair, several novel analogs based on the same pentapeptide backbones have been developed (entries 7–13, Table 1; Figure 3). Here, the major innovation lies in the design of an alternative linker structure between the peptide core and the radiolabel/fluorescent dye. Compared to the parent peptides [^68^Ga]Pentixafor and [^177^Lu]Pentixather, the compounds bearing the novel r-a-ABA-linker (entries 8 and 9, Table 1) or variants thereof (entries 10 and 11, Table 1) show particularly high hCXCR4 affinities (improvement by a factor of 2–10 compared to [^68^Ga]Pentixafor) and enhanced internalization, indicative of an increasingly agonistic profile [62]. Of note, the use of the iodo-Tyr^3^-containing Pentixather-backbone decreases the selectivity of the ligands for the human CXCR4 receptor (entries 8 and 9, Table 1), providing tracers with decreased species selectivity and thus potential applicability for studying CXCR4-related pathologies in murine models. As observed for the cyclam-based tracers (entries 1–5, Table 1, and discussed in detail above), this increase in affinity to murine CXCR4 is consistently accompanied by an increasing tracer uptake in mCXCR4-expressing tissues such as lung, liver, spleen and bone marrow. Thus, in analogy to, e.g., [^64^Cu]CB-bi-cyclam (entry 1, Table 1), the high but blockable hepatic activity level observed for [^177^Lu]DOTA-r-a-ABA-iodoCPCR4 is mCXCR4-specific and not the result of an unfavorable pharmacokinetic profile with hepatobiliary clearance of the ligand. The same applies to [^99m^Tc]PentixaSPECT (entry 11, Table 1), for which relatively high hepatic uptake was observed in mice, but not in patients (see Figure 4), further corroborating the relevance of mCXCR4-mediated tracer uptake in mouse liver and the danger of misinterpreting it as “unfavorable tracer pharmacokinetics”.

In contrast, [^125^I]CPCR4.3 (entry 7, Table 1; Figure 3), with a log P = 0.51, truly shows predominant clearance via the liver and intestines, in addition to mCXCR4-driven liver uptake, and thus is not suited for in vivo imaging purposes [75]. However, due to its excellent affinity to both human and mouse CXCR4, it may serve as an excellent preclinical tool for the sensitive detection of low levels of CXCR4 expression in vivo, e.g., via ex vivo autoradiography [76].

Another highly promising approach is the use of the cyclic peptide Ac-Arg-Ala-[D-Cys-Arg-2-Nal-His-Pen]-COOH [101] as a scaffold for tracer development. The corresponding [^68^Ga]NOTA-AMBS-Ahx-conjugated analog (entry 14, Table 1; Figure 3) shows excellent CXCR4-targeting in vitro and in vivo, and despite lower absolute uptake in Daudi xenografts (ca. 50% of the uptake found for [^68^Ga]Pentixafor [102]), improved tumor/background ratios due to particularly low background accumulation [80].

Intense efforts have also been directed towards the development and optimization of imaging probes/theranostic pairs based on the LY2510924 cyclic octapeptide sequence (entries 15–20, Table 1; Figure 2 and Figure 3). Pioneered by the development of [^67^Ga]FRM001 [81], a series of closely related ligands for CXCR4-targeted theranostics (entries 16 and 17, Table 1) and PET imaging (entries 18–20, Table 1) were designed and progressively optimized [82,83]. Of note, in a direct comparative biodistribution study, tracer accumulation in Daudi xenografts and in kidneys was identical for [^18^F]BL-08 and [^68^Ga]Pentixafor at 1 and 2 h p.i. However, due to substantially lower background accumulation of [^18^F]BL-08 in all other organs, tumor/background ratios and imaging contrast were superior for the radio-fluorinated LY2510924 analog [83]. In contrast, [^64^Cu]NOTA-CP01, in which only a very short linker between the peptide core and the radiolabel was used, shows less-promising results [84].

Furthermore, three highly interesting and diverse protein-based approaches have been pursued (entries 22–24, Table 1 [68,86,87]), all of which showed some promise for imaging of CXCR4 expression in vivo, albeit with the common limitations associated with using macromolecules for imaging applications (slow clearance kinetics, high non-specific background accumulation).

Overall, it can be anticipated that in the “race for clinical application”, the novel peptide-based tracers with their highly optimized pharmacokinetics and excellent CXCR4-targeting properties will certainly start from the pole position, closely followed by cyclam-based candidates such as [^64^Cu]CB-bi-cyclam. The increasing insight into CXCR4 receptor–ligand interactions and expanding structure–activity relationships will certainly also support the development of improved ligands for more complex applications, such as hybrid (radiolabeled/fluorescent) tracers for intraoperative guidance [103].

## 6. Conclusions

Based on its eminent role in both tumor biology as well as in inflammation, CXCR4 is a molecular imaging target of utmost interest and with high potential as a predictive marker for disease outcome in the context of the aforementioned pathologies.

Especially in inflammation imaging, the current first “proof-of-concept” data in patients need to be further validated in prospective clinical trials. However, the existing data clearly show the high potential of this approach for specifically visualizing inflammation and its resolution over time in a broad spectrum of inflammatory diseases. CXCR4-targeted imaging may thus serve as a tool for non-invasive therapy control after anti-inflammatory treatments and for providing personalized anti-inflammatory treatments to improve disease outcome. Additionally, CXCR4-directed imaging of immune cells holds great promise to address some other urgent clinical questions, such as the need for an imaging tool to quantify the influx of CXCR4-positive immune cells into immunologically “cold” tumors after checkpoint inhibition therapy. First preclinical data hint towards a general feasibility of such an approach [88]. This, however, needs careful in-depth validation, also including a careful selection of cancer patients who might profit from such an approach (e.g., cancers with negligible tumoral CXCR4 expression).

With the continuous emergence of additional, improved radiopharmaceuticals, a further expansion of the scope of CXCR4-targeted imaging and therapy can be anticipated. This does not only encompass an expansion of the preclinical and clinical scope towards other CXCR4-driven inflammatory pathologies, but also a broadening of the spectrum of applicable imaging modalities, now including SPECT, optical imaging (e.g., endoscopy) or a combination of both (hybrid surgical guidance).

## Figures and Tables

**Table 1:** Summary of recently developed novel CXCR4-targeted imaging agents (2019–2021). If not stated otherwise in the table, all internalization and biodistribution data were obtained at 1 h incubation or at 1 h p.i. respectively, and internalization studies were performed in the cell line used for tumor induction.

Entry	Compound	Affinity [IC_50_, nM]	Internalized [% of Total Cellular Activity]	Tumor Uptake [%iD/g]	Liver [%iD/g]	T/Muscle Ratio	Ref.
1	[^64^Cu]CB-bicyclam	8 (hCXCR4) ^b^	n.d.	SUV_max_: 7.4 ± 1.8 (U87.CXCR4)	13.8	23.6 ± 2.7	[69]
		2 (mCXCR4) ^a^		SUV_max_: 0.8 ± 0.1 (U87)		3.0 ± 0.5	
2	[^18^F]MCFB	111 ± 4 ^a^	~40	3.3 ± 0.9 (U2932)	63 ± 5	4.0 ± 0.8	[70]
				1.8 ± 0.1 (SUDHL-8)	52 ± 3	2.1 ± 0.3	
3	[^99m^Tc]AMD3465	n.d.	n.d.	2.1 ± 0.4 (MCF-7)	25 ± 7	4.7	[71]
4	[^18^F]RPS-534	218 ± 38 ^c^	~7 (2 h)	7.2 ± 0.3 (PC3-CXCR4)	19.1 ± 0.4	42.4 ± 0.1	[72]
5	[^76^Br]HZ270-1	6.7 ± 0.7 ^c^	n.d.	9.5 ± 1.3 (U87.CXCR4, 24 h)	7.6 ± 1.3 (24 h)	n.d.	[73]
6	[^18^F]5	6.9 ^d^	n.d.	4.0 ± 0.3 (SCCHN, 1.5 h)	1.2 ± 0.1 (90 min)	25	[74]
7	[^125^I]CPCR4.3	5.4 ± 1.5 (hCXCR4) ^e^	68 ± 3 (MCF-7)	n.d.	21.2 ± 2.9	n.d.	[75,76]
		4.9 ± 1.7 (mCXCR4) ^f^					
8	[^177^Lu]DOTA-r-a-ABA-CPCR4	1.5 ± 0.1 (hCXCR4) ^e^	65 ± 6 (Chem1)	18.3 ± 3.7 (Daudi)	11.9 ± 1.6	413 ± 100	[62]
		182 ± 26 (mCXCR4) ^f^					
9	[^177^Lu]DOTA-r-a-ABA-iodoCPCR4	1.7 ± 0.6 (hCXCR4) ^e^	91 ± 4 (Chem1)	17.2 ± 2.0 (Daudi)	27.1 ± 1.9	226 ± 36	
		49 ± 1 (mCXCR4) ^f^					
10	[^125^I]MK007	10.2 ± 4.0 ^e^	n.d.	1.1 ± 0.1 (Jurkat)	35.3 ± 1.0	~2	[77]
11	[^99m^Tc]PentixaSPECT	10.2 ± 2.4 ^g^	95 (Chem1)	8.6 ± 1.3 (Jurkat)	7.7 ± 0.7	29 ± 6	[78]
12	[^99m^Tc]HYNIC-L	K_d_ 3.3 ± 0.4 (DU-4475)	~9	3.2 ± 0.9 (DU-4475)	2.0 ± 0.4	n.d.	[79]
13	[^177^Lu]DOTA-HYNIC-L	K_d_ 3.2 ± 0.4 (DU-4475)	~9	4.2 ± 1.1 (DU-4475)	2.3 ± 0.5	n.d.	
14	[^68^Ga]**5**	15.6 ± 4.2 ^e^	91 (Chem1)	7.9 ± 1.4 (Daudi)	0.36 ± 0.01	115 ± 48	[80]
15	[^67^Ga]FRM001	2.3 ± 0.5 ^a^	~15	12.0 ± 2.0 (CCRF-CEM, 4 h)	16.1 ± 2.7 (4h)	112	[81]
16	[^68^Ga]BL-01	21.2 ± 16 ^a^	n.d.	10.2 ± 2.6 (Daudi)	7.1 ± 1.3	23 ± 4	[82]
17	[^177^Lu]BL-01	7.1 ± 1.7 ^a^	n.d.	14.0 ± 1.1 (Daudi)	10.3 ± 0.9	32 ± 3	
18	[^18^F]BL-08	11.6 ± 7.0 ^a^	n.d.	7.6 ± 1.4 (Daudi)	0.62 ± 0.02	108 ± 25	[83]
19	[^18^F]BL-09	13.4 ± 2.3 ^a^	n.d.	6.6 ± 2.1 (Daudi)	0.56 ± 0.09	83 ± 19	
20	[^64^Cu]NOTA-CP01	1.6 ± 1.0 ^h^	n.d.	SUV_max_: 1.3 ± 0.2 (EC109, 6 h)	SUV_max_: ~3.5 (6 h)	15.4 ± 3.0 (6 h)	[84]
21	[^99m^Tc]T140 analog	1.9 ^a^	negligible	0.5 ± 0.1 (U87.CXCR4, 2 h)	22.7 ± 5.0 (2 h)	~2 (2 h)	[85]
22	[^64^Cu]DOTA-vMIP-II	n.d.	n.d.	~4.5 (aortic plaque)	n.d.	~3	[68]
23	[^64^Cu]MCo-CVX-6D	0.07 ± 0.02 (FRET)	n.d.	5.7 ± 0.5 (U87.CXCR4, 24 h)	23.3 ± 2.1 (24 h)	19.9 ± 4.7	[86]
24	[^64^Cu]Ubiquitin	n.d.	n.d.	1.6 ± 0.6 (4T1, 2 h)	~4.8 (2 h)	8.5 ± 2.3 (2 h)	[87]

^a^ competitive binding assay using [^125^I]SDF-α as radioligand; ^b^ calcium flux assay; ^c^ competitive binding assay using [^61^Ga]pentixafor as radioligand; ^d^ fluorescence-based competitive binding assay against TN14003; ^e^ competitive binding assay using [^125^I]FC-131 as radioligand; ^f^ competitive binding assay using [^125^I]CPCR4.3 as radioligand; ^g^ competitive binding of [^99m^Tc]PentixaSPECT vs cold FC-131; ^h^ competitive binding of [^64^Cu]NOTA-CP01 vs unlabeled precursor.

## Data Availability

No new data were created or analyzed in this review. Data sharing is not applicable to this article.
